# An Assessment of the Effect of Green Synthesized Silver Nanoparticles Using Sage Leaves (*Salvia officinalis* L.) on Germinated Plants of Maize (*Zea mays* L.)

**DOI:** 10.3390/nano9111550

**Published:** 2019-10-31

**Authors:** Karel Sehnal, Bozena Hosnedlova, Michaela Docekalova, Martina Stankova, Dagmar Uhlirova, Zuzana Tothova, Marta Kepinska, Halina Milnerowicz, Carlos Fernandez, Branislav Ruttkay-Nedecky, Hoai Viet Nguyen, Augustine Ofomaja, Jiri Sochor, Rene Kizek

**Affiliations:** 1 Department of Viticulture and Enology, Faculty of Horticulture, Mendel University in Brno, Valticka 337, CZ-691 44 Lednice, Czech Republic; karelsehnal15@gmail.com (K.S.); bozena.hosnedlova@post.cz (B.H.); docekalova@preventionmedicals.cz (M.D.); MartStan@seznam.cz (M.S.); uhlirova@preventionmedicals.cz (D.U.); ztothova984@gmail.com (Z.T.); brano.ruttkay@seznam.cz (B.R.-N.); jiri.sochor@mendelu.cz (J.S.); 2 Department of Human Pharmacology and Toxicology, Faculty of Pharmacy, University of Veterinary and Pharmaceutical Sciences Brno, Palackeho 1946/1, 612 42 Brno, Czech Republic; 3 Department of Research and Development, Prevention Medicals, Tovarni 342, 742 13 Studenka-Butovice, Czech Republic; 4 Department of Food and Feed Safety, Veterinary Research Institute, Hudcova 70, 621 00 Brno, Czech Republic; 5 Department of Biomedical and Environmental Analyses, Faculty of Pharmacy with Division of Laboratory Medicine, Wroclaw Medical University, Borowska 211, 50-556 Wroclaw, Poland; zalewska.m@gmail.com (M.K.); halina.milnerowicz@umed.wroc.pl (H.M.); 6 School of Pharmacy and Life Sciences, Robert Gordon University, Garthdee Road, Aberdeen AB10 7QB, UK; c.fernandez@rgu.ac.uk; 7 Research Center for Environmental Monitoring and Modeling, VNU University of Science, Vietnam National University, 334 Nguyen Trai Street, Hanoi 100000, Vietnam; nguyenviethoai@hus.edu.vn; 8 Biosorption and Wastewater Treatment Research Laboratory, Department of Chemistry, Faculty of Applied and Computer Sciences, Vaal University of Technology, P. Bag X021, Vanderbijlpark 1900, South Africa; augustineo@vut.ac.za

**Keywords:** green synthesis, thiol compounds, phytotoxicity, phyto-nanotechnology, plant physiology

## Abstract

AgNPs have attracted considerable attention in many applications including industrial use, and their antibacterial properties have been widely investigated. Due to the green synthesis process employed, the nanoparticle surface can be coated with molecules with biologically important characteristics. It has been reported that increased use of nanoparticles elevates the risk of their release into the environment. However, little is known about the behaviour of AgNPs in the eco-environment. In this study, the effect of green synthesized AgNPs on germinated plants of maize was examined. The effects on germination, basic growth and physiological parameters of the plants were monitored. Moreover, the effect of AgNPs was compared with that of Ag(I) ions in the form of AgNO_3_ solution. It was found that the growth inhibition of the above-ground parts of plants was about 40%, and AgNPs exhibited a significant effect on photosynthetic pigments. Significant differences in the following parameters were observed: weights of the caryopses and fresh weight (FW) of primary roots after 96 h of exposure to Ag(I) ions and AgNPs compared to the control and between Ag compounds. In addition, the coefficient of velocity of germination (CVG) between the control and the AgNPs varied and that between the Ag(I) ions and AgNPs was also different. Phytotoxicity was proved in the following sequence: control < AgNPs < Ag(I) ions.

## 1. Introduction

**Figure 1:**
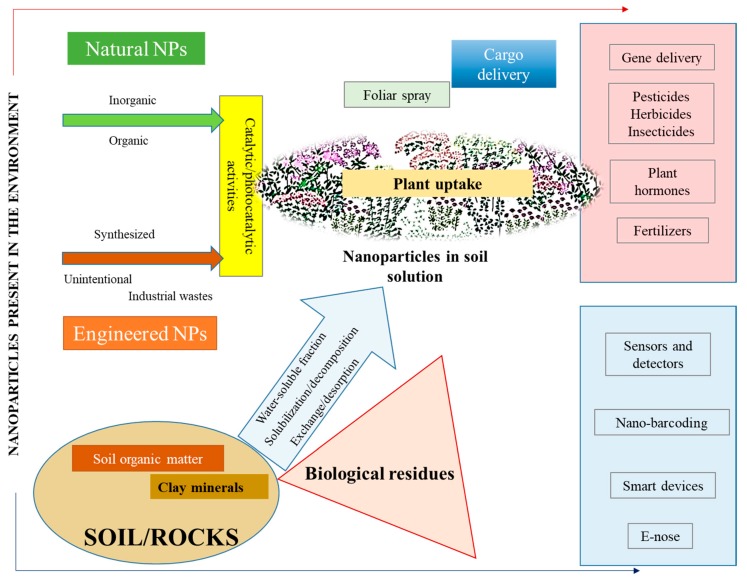
A simplified scheme of the presence and movement of nanoparticles in the environment. Nanoparticles (NPs) get into the environment in a natural way (natural nanoparticles) in the inorganic (volcanic dust, etc.) or possibly organic form (cellular debris), as well as due to human activity (engineered nanoparticles). Such nanoparticles are purposefully synthesized or generated by unwanted processes or as a part of industrial waste. These particles can bind to the soil. From there they are mobilized into the environment as a water fraction, in which they can be solubilized. Thus, the particles can enter the plants through the root system [14].

Nowadays, nanomaterials have a wide range of applications in different fields such as biotechnology, physics, chemistry, and electronics [1,2,3,4,5,6]. Various nanoparticles are the basis for a new innovative method for delivering molecules [7]. The number of nanotechnology products is exponentially growing worldwide. In 2013, there were more than 1650 nanotechnology products, especially in biomedical and agriculture sectors [8,9]. In the field of agriculture and plant applications, the maximum focus was on disease prevention, nano-fertilizers, and nanosensors for the detection of soil deficiencies [10]. Nanoparticles in connection with plants could in the future become very important in bioelectronics as electronic plant technologies (e-Plant concept) [11]. Due to the increased use of nanomaterials, there are growing concerns over their potential adverse impacts on the eco-environment [12]. So far, the current environmental impact of nanomaterials is still not reliably documented and described, including how to best verify this impact [13]—see in Figure 1.

Silver nanoparticles (AgNPs) can be prepared by physical, chemical, [15] and biological [16,17,18,19] synthesis, and can be synthesized in a variety of shapes and sizes, which opens the way for other applications [20]. The green synthesis of AgNPs brings completely new unique properties for prepared nanoparticles. Many different modifications of AgNPs using various medicinal and agricultural plants *(Alium sativum, Azadirachta idica*, *Curcubita maxima*, *Podophyllum hexandrum, Solanum trilobatum, Rosa indica*) have been prepared [21]. A very important group of modifications of AgNPs is the use of *Curcuma longa* due to its antiproliferative properties [22]. Green synthesis of nanoparticles enables their use for many antimicrobial purposes, as well as in anticancer, anti-inflammatory, and wound treatment applications [19]. In the process of preparing nanoparticles, natural substances are used to reduce metal salts, and no other reducing agents or stabilizing agents are applied. The nanoparticles obtained show very good biological properties [23]. AgNPs produced by photosynthesis can exhibit both antibacterial [24] and antitumor effects [25,26]. In addition, in a study of Dakshayani et al., anticoagulant and antiplatelet properties of AgNPs prepared from *Selaginella bryopteris* were shown [27], and in a study of Almeida et al., both high angiogenic and osteogenic activities of AgNPs synthesized using *Hancornia speciosa* was also observed [28]. 

Biosynthesis using plant extracts excluding the use of chemical agents associated with environmental toxicity is considered environmentally friendly [29,30]. Plants prevent the presence of heavy metal ions by synthesizing thiol compounds such as glutathione and phytochelatin. Thiol compounds form complexes with metal ions that are subsequently transported to the vacuole where they are inactivated and their toxicity decreases significantly [31]. Plants can absorb nanoparticles by all exposure routes including soil, water, and air [32]. An important aspect is the risk assessment of nanoparticles and the understanding of nanoparticle interactions with plants as essential components of all ecosystems [33,34]. Application of nanoparticles to a plant can result in the modification of gene expression and alteration of genetic pathways, which ultimately affects the growth and development of that plant [35]. In a recent study [36], the effect of foliar applied nanoparticles was studied. AgNPs have been shown to interfere with the nitrification activity and possibly affect nitrogen oxide emissions [37]. 

It has also been reported that nanoparticles can result in reactive oxygen species (ROS) generation in the plant [38,39,40]. The mechanism associated with nanoparticle-induced ROS formation varies across different types of nanoparticles, and the core cellular mechanism related to ROS production remains unexplained. Most metal nanoparticles may provoke free-radical-facilitated toxicity via Fenton-type reactions [41]. However, some research studies have demonstrated the positive effects of nanoparticles on higher plants in addition to their negative effects [39,42,43]. Silver nanoparticles (AgNPs) have enjoyed great popularity in commercial production in recent years; however, studies on their toxic effects are still limited [44,45]. A number of experimental works have been conducted on the onion (*Allium cepa*) plant model due to the ease of studying cell division and chromosomal changes. The interaction of silver ions with DNA is mainly known for guanines, where cross-linking can then occur, and the processes of replication and transcription can be prevented. Therefore, genotoxicological studies are important for such types of nanoparticles. A recent study investigated the cytotoxic and genotoxic potential effect of the AgNPs and/or ZnNPs on root cells of *A. cepa* [45,46]. These authors reported that the AgNPs penetrated the roots of the plant and affected the mitotic index, germination, nuclear abnormality, and micronucleus index in meristematic cells [45,47]. 

**Figure 2:**
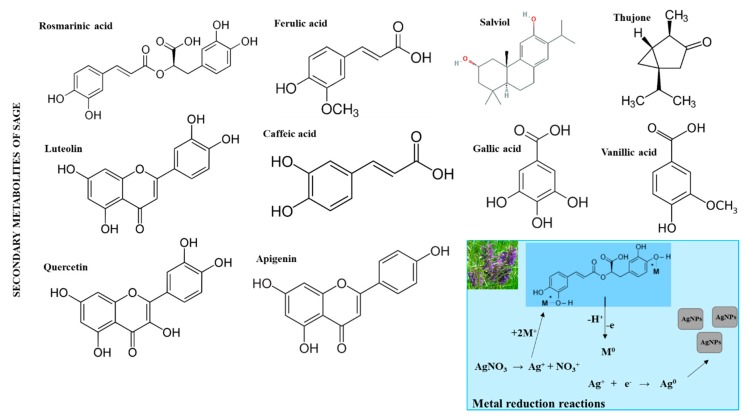
Biologically important secondary metabolites found in *Salvia officinalis* at high concentrations. Antibacterially active molecules such as salviol and thujone have been identified. In inset: A proposed scheme for reduction of silver ions in the presence of phenolic compounds is also shown. The resulting reduced ions subsequently form aggregates in the form of nanoparticles.

Sage (*Salvia*) is known worldwide in traditional medicine [48]. This plant is found in the temperate, subtropical, and tropical areas. In addition to its ethnobotanical importance, some species of sage such as *Salvia officinalis* (sage, common sage), *S. sclarea* (clary sage), *S. lavandulifolia* (Spanish sage), *S. miltiorrhiza* (danshen), and *S. hispanica* (chia) are used as a food and due to the content of essential oils, have become popular in traditional medicine [48]. Sage is known for its substances, especially phenolic compounds with the highest content of caffeic acid, vanillic acid, ferulic acid, luteolin, apigenin, quercetin, rosmarinic acid and their derivatives [49,50,51]—(Figure 2). 

Recently, chemical characteristics of various sage species were summarized by Er et al. [52]. The *Salvia* genus includes around 900 species, of which three, *S. officinalis*, *S. lavandulaefolia* and *S. miltiorrhiza,* are particularly remarkable due to their beneficial effects on behavioural function [53]. Thanks to modern extraction techniques, new components with extraordinary therapeutic effects, mainly in the context of neurodegenerative diseases and various cancers, have been discovered. These include pro-cholinergic (including cholinesterase inhibition), anti-inflammatory, antioxidant and estrogenic properties [54]. Thus, the extracts obtained from them exhibit significant antibacterial and antioxidant activities [55]. Species of sage have a long-standing reputation in European medical herbalism, including their use for memory enhancement. It has been reported that administration of sage extracts with proven cholinergic properties improved cognitive function in young adults [54]. In addition, anticancer effects on mammary carcinoma, leukaemia cells [49,56,57,58] and antiviral effects on HIV [59] have been demonstrated. 

This study deals with the effect of different concentrations of AgNPs synthesized through a green approach (using sage extract) in comparison to that of Ag(I) ions on germinated plants of maize (*Zea mays*). The germination rate was assessed at 24, 48, and 96 h. After 96 h, the experiment was terminated and the roots were evaluated morphologically (length, colour), microscopically (changes in root structure) and biochemically (total protein). The experiment was evaluated using three independent repetitions (*n* = 150).

## 2. Materials and Methods 

### 2.1. Chemicals and Material

Silver nitrate, methanol, NaCl, 2,2-diphenyl-1-picrylhydrazyl (DPPH), gallic acid, caffeic acid, Folin–Ciocalteu reagents and other chemicals were purchased from Merck (Darmstadt, Germany) at a purity >99%. All chemicals that we used for gel electrophoresis were purchased from VWR (Hamburg, Germany). All plastic materials used (tubes, tips) in this study were purchased from Eppendorf (Hamburg, Germany). Dried sage leaves (*Salvia officinalis* L., *Lamiaceae*) were purchased from Valdemar Grešík—Natura s.r.o. (Decin, Czech Republic). Working standard solutions were prepared daily by dilution of the stock solutions.

### 2.2. Deionised Water, pH, and Ion Analysis

Deionised water was prepared by using a reverse osmosis equipment Aqual 25 (Brno, Czech Republic), and was further purified by using an ELGA apparatus equipped with a UV lamp (Lane End, UK). The resistance was 18 MΩ and the pH was measured using a pH meter (WTW, Prague, Czech Republic).

### 2.3. Preparation of the Sage Plant Extracts

Plant materials were ground and macerated for extraction. Fifty grams of each sample were weighed into one-litre Erlenmeyer flasks, and then 500 mL of solvents with varying polarities (methanol, ethanol, diethyl ether and hexane) were added to the plant samples. Extraction was carried out by shaking at room temperature for 72 h. After filtration through filter paper (Whatman No. 4, Madestone, UK), the residue was re-extracted twice, and then the combined extracts of every sample were evaporated at room temperature and dried in desiccators under vacuum to a constant weight [60]. 

### 2.4. Synthesis of AgNPs

We used the procedure from our previous paper with minor modification [61]. Briefly, the mixture was homogenized by milling to 1–2 mm particles, then it was extracted and subsequently stirred in ultrapure water (80 °C, 60 min) at a ratio of 5 dry weight (DW) g/100 mL, *v/w*. The leachate was further centrifuged (30 min, 4000 g) and then mixed with 0.1 M AgNO_3_ (1:1). The solution was stirred on a magnetic stirrer (80 °C, 24 h). The particles were prepared by precipitation with methanol (1:1) and left on a magnetic stirrer (60 min). After purification, the supernatant was removed and the particles were allowed to dry in a dryer DRY-Line (24 h, 60 °C, VWR, Hamburg, Germany).

### 2.5. Germination of Maize Seeds

Seeds were disinfected with 10% sodium hypochlorite solution for 30 s. After the treatment, 50 seeds were germinated on five layers of filter paper in on 25 × 25 cm plastic box. The boxes were covered to prevent the loss of moisture by evaporation under laboratory conditions (25 ± 1°C) for 5 days. Seeds were considered germinated when they exhibited a radicle extension of ≥ 3 mm. Every 24 h we determined the following germination parameters: Final germination percentage (FGP) = Ng/Nt × 100, where Ng is a total number of germinated seeds and Nt is a total number of seeds evaluated. Also, mean germination time (MGT) and the germination index (GI) were calculated. GI = number of germinated seeds/days of the first count and number of germinated seed/days of the final count. The coefficient of velocity of germination (CVG) was determined by a mathematical manipulation: CVG = ΣN_i_/ΣN_i_T_i_ × 100. Mean daily germination (MDG) which is the index of daily germination, was calculated from the following equation: MDG = FGP/d, where FGP is the final germination percentage and d represents the days to the maximum final germination [62].

### 2.6. Planting

Plant cultivation was based on our previous works [31,63,64,65,66]. Briefly, seeds of maize (*Zea mays*) of the Silen variety were sprouted on cellulose wadding in cultivation boxes (Batist Medical a.s., Cerveny Kostelec, Czech Republic) in an amount of 10 × 10 seeds and were watered by tap water (250 mL, conductivity of 480 µS/cm, pH 6.5). The boxes were left for one week at room temperature (25 °C). For our experiment, 7-day-old maize seedlings were selected. The plants were chosen to be uniform in size. AgNO_3_ and AgNPs were applied at 1, 50, 150 mg/L. Preparation of nanoparticles for the application was as follows: homogenization was achieved by ultrasound, AgNPs (2, 100, 300 mg) were added into the 2 mL Eppendorf tubes and the tubes were supplemented with ultrapure water to their 2 mL volumes. Then, three various concentrations (1, 50, 150 mg/L) of Ag(I) ions were prepared. Silver nitrate (Ag(I) ions) was used as a positive control. Silver nitrate (3.15, 157.48, 472.44 mg) was added into the 2 mL Eppendorf tubes. Distilled water was used as a negative control. 

### 2.7. Greenhouse Conditions

The experiment was conducted in a climate-controlled greenhouse. The nominal maximum temperature in the greenhouse was set at 25 °C. The seedlings were, after this time, placed into a hydroponic system with 3 L of cultivation solution and with a light of 36 W/865, FAR of 100 µmol/m^2^/s with light/dark intervals of 12 h/12 h. 

### 2.8. Harvesting Description

The plants were harvested after 48 h of growth. Aboveground plant tissue was removed from the hydroponic system, and mass was immediately measured. The plants were then divided according to tissue type, stem, leaves, and pods, and were weighed separately. Leaves and pods were photographed for size analysis. Masses were recorded before and after drying to determine the dry mass and water content. The root system of each plant was removed from the hydroponic system followed by rinsing (1 min, three times) in 1 mM EDTA and deionized H_2_O. The root system was allowed to air dry (~20 min) before weighing for wet mass. Plants tissue were stored (4 °C and −80 °C) for future analysis.

### 2.9. Photosynthetic Dyes Analysis

Analysis of photosynthetic dyes: To determine the number of chlorophylls, 1 g of the above-ground portion of the plant was weighed, placed in a mortar and ground with sea sand. Then, 1 mg of magnesium oxide was added and, after a short period of grinding, 10 mL of acetone was added. The sample was filtered through filter paper (with 100 µm pore size). The filtered sample was refilled with acetone to 25 mL volume in a volumetric flask. The extracted chlorophyll was diluted into a 2 mL glass cuvette with acetone at a ratio of 1:9. Chlorophyll measurements were performed in the range 350–650 nm with a scan of 2 nm. 

### 2.10. Plant Growth Metrics

The plants were always checked at 18:00 UTC for 24 h. Physiological and morphological evaluation of plants: Stem (length, number of leaves) and root (length of longest and shortest root, number of roots) morphology of the plants was observed (*n* = 5). The amounts of chlorophyll, silver, protein and phenolic compounds were determined (*n* = 3). In addition, quantification and analysis of the morphological parameters of the root system (microscopic and photographic evaluation) were performed. For determining the fresh weight (FW), plants were weighed (Boeco, Hamburg, Germany) immediately after removal from the hydroponic system and were placed on filter paper (*n* = 4). For the determination of dry weight (DW), the plants were divided into smaller parts and left for 48 h at 60 °C (*n* = 5). 

### 2.11. Morphological and Anatomical Studies

The morphological changes in the root, stem and leaf of maize treated with silver ions and AgNPs were documented up to 5 days at 1-day intervals. To evaluate the effect induced by AgNO_3_ and AgNPs, the plants were photographed (Canon, Full HD 20.3 Mpx, Tokyo, Japan). Microscopic analysis was performed using a computer connected with the microscope VisiScope (VWR, Hamburg, Germany) allowing photo collection (10 MPx) on a PC. Image analysis was performed by ColorTest (Prevention Medicals, Studenka, Czech Republic). 

### 2.12. Quenching of Radicals

Characterization of the nanoparticle surface was performed by methods previously optimized [12,13,14,15,16,17,19,67]. Ferric Reducing Antioxidant Power (FRAP) was based on the reduction of 2,4,6-tripyridyl-s-triazine complex (TPTZ) with FeCl_3_·6H_2_O, and the absorbance was measured at 605 nm. The radical of 2,2′-azino-bis(3-ethylbenzothiazoline-6-sulfonic acid)-(ABTS, 7 mM) and potassium peroxodisulfate (5 mM) were mixed in water. The solutions were then prepared by diluting with water in a ratio of 1:9 *v*/*v*, stored for 12 h in the dark at 4 °C prior to using, and the absorbance was measured at 660 nm. The DPPH (2,2-diphenyl-1-picrylhydrazyl) method is based on quenching the color of the radical whose absorbance is measured at 510 nm. 

### 2.13. Total Phenolic Content (TPC) Determination

Total phenolic contents in the extracts were determined by the Folin-Ciocalteau reagent (316 µL of reagent was mixed with 4 µL of the sample), in which the mixture was left at 25 °C for 5 min followed by the addition of 80 µL of 6% (*w*/*v*) Na_2_CO_3_. The sample was left for 90 min at 25 °C and the absorbance was measured at 670 nm. A standard curve was prepared by using different concentrations of gallic acid and the absorbances were measured at 670 nm. The total phenolic content was expressed as mg of gallic acid equivalents/g dry extract weight (mg GAE/g DW). Analyses were done in triplicate [67].

### 2.14. Total Flavonoid Content Determination 

The total flavonoid content was determined by the following procedure: 0.5 mL of the sample was mixed with 1.5 mL of methanol, 0.1 mL of 10% aluminium chloride, 0.1 mL of 1 M potassium acetate and 2.8 mL of water. The resulting solution was left at 25 °C for 30 min and the absorbance was measured at 415 nm. The quercetin calibration dependence was prepared to determine the concentration of quercetin [68]. 

### 2.15. Total Protein (Biuret Method and Pyrogallol Red-Molybdate Method)

To an aliquot (50 µL) of the supernatant diluted to 1 mL with extraction buffer, 5 mL of biuret and/or pyrogallol red was added and mixed thoroughly. The absorbance was read at 510 and/or 520 nm reagent blank. The amount of protein was calculated using standard prepared with different concentrations of soy protein.

### 2.16. Pseudo-Peroxidase Assay

AgNPs (10 µL) were pipetted into a plate (Gama group, Ceske Budejovice, Czech Republic) to 200 µL of substrate solution. The substrate solution was composed of 915 µL of 0.5 M acetate buffer (pH 4.0) and 100 µL of 5 mM 3,3′,5,5′-tetramethylbenzidine (TMB) (in 100% DMSO) along with 85 µL H_2_O_2_ (30%). Subsequently, 200 µL of substrate solution with TMB was added to the AgNPs which remained in the well. After 30 min incubation (at 25 °C), with shaking at 2 min intervals, the color development appeared. The color development appeared after incubation. The absorbance was recorded at 620 nm. To evaluate the pseudo-peroxidase activity, the absorbance value was used at 30 min. As a control sample for assessment of pseudo-peroxidase activity, 10 µL of 1 U horseradish peroxidase (HRP) and 200 µL of substrate solution were used per reaction.

### 2.17. Silver Sample Collection-Electrochemical Measurement

Electrochemical measurements were performed using an Autolab analyser (Metrohm, Herisau, Switzerland). The three-electrode system consisted of the carbon paste working electrode and or carbon tip, an Ag/AgCl/3 mol/dm^3^ KCl reference electrode and a carbon counter electrode. The differential pulse voltammetry parameters were as follows: initial potential −0.1 V, end potential 0.8 V, modulation amplitude 25 mV and step potential 0.5 mV. All experiments were carried out at 25 °C. Acetate buffer (0.2 mol/dm^3^, pH 5.0) was used as the supporting electrolyte. The raw data were treated using the Savitzky and Golay filter (level 2) and the moving average baseline correction (peak width 0.03) of the NOVA software. The carbon paste was made of 70% graphite powder (Merck, Darmstadt, Germany) and 30% mineral oil (Merck, Darmstadt, Germany; free of DNase, RNase, and protease). The carbon paste was housed in a Teflon body having a 2.5 mm diameter of the active disk surface. The electrode surface was polished before each determination with soft filter paper prior to measurement.

### 2.18. Electron Microscopy of the AgNPs

Surface morphology of the nanoparticles was investigated with field emission scanning electron microscopy (FESEM) using an operating voltage of 10 kV in the SEM (Zeiss, Oberkochen, Germany) instrument. The surface charging effect was minimized by coating the samples with gold on copper stubs with a coating instrument. Transmission electron microscopy (TEM) and higher resolution TEM (JEOL, Tokyo, Japan) were determined using a copper stub with carbon glue and coated with gold before analysis. The samples for TEM and HRTEM were placed in vials containing absolute ethanol and ultrasonicated for 10 min. Thereafter, holey/lacey carbon grids (10 µm) were dipped into the vials containing the ultrasonicated samples and dried before microstructural determination.

### 2.19. Absorbance Measurements

Spectrophotometry: a UV-Vis UV-3100PC, VWR (Hamburg, Germany) single-beam spectrophotometer was used to record the UV-Vis spectra. The Vis spectrum was measured every 2 nm in the range of 350–700 nm in plastic cuvettes with an optical path of 1 cm. An Infinite F50 (Tecan, Mannedorf, Switzerland) was used for measurement on a polystyrene microtiter plate (Gama Group a.s., Ceske Budejovice, Czech Republic). Automated spectrometric measurements: BS-300 chemical analyser from Mindray (Shenzhen, China), cuvettes 5 × 6 × 30 mm, optical path 5 mm and a volume of the reaction mixture in the cuvette 180–500 µL. Photometric detector measuring at wavelengths: 340, 405, 450, 510, 546, 578, 630, and 670 nm. Reagents and samples were placed on the cooled sample holder (4 °C) and automatically pipetted directly into plastic cuvettes. Incubation proceeded at 37 °C. The mixture was consequently stirred. The washing steps by distilled water (18 mΩ) were done in the midst of the pipetting. Apparatus was operated using the software BS-300 (Mindray, Shenzhen, China) and LADYS (Prevention Medicals, Studenka, Czech Republic).

### 2.20. Zetasizer Analysis of Nanoparticles

The size distribution (i.e., the hydrodynamic diameter, DH) was determined by dynamic light scattering (DLS) using the Zetasizer Nano ZS ZEN3600 (Malvern Instruments, Malvern, UK) with a detection angle of 173° in optically homogeneous square polystyrene cells. The samples were diluted hundredfold with deionized water. All measurements were performed at 25 °C. Each value was obtained as an average of 5 runs with at least 10 measurements. Version 7.10 of the Zetasizer Software was applied for data evaluation. The particle charge (ζ-potential) was measured by the microelectrophoretic method using a Malvern Zetasizer Nano ZS ZEN3600 (Malvern Instruments, Malvern, UK). All the measurements were performed at 25 °C in polycarbonate cuvettes. Each value was obtained as an average of 5 subsequent runs of the instrument with at least 20 measurements.

### 2.21. Data Treatment and Descriptive Statistics

Experimental work was performed using at least three independent experiments. Each sample in the experiments was analysed at least five times. The obtained data presented in this paper are the average values. No experimental points were excluded from the proposed experimental study. All the obtained data were stored in the Qinslab database (Prevention Medicals, Studenka, Czech Republic). If possible, data were processed and evaluated mathematically and statistically in the Qinslab database. The results were expressed as the mean ± standard deviation (SD). Photos were processed by the ColorTest program, which assigns an intensity to the individual pixels of the studied image in a given colour area [18]. For preparing the publication, the data were processed using MICROSOFT software (Redmont, DC, USA).

## 3. Results and Discussion

With increasing interest in nanotechnology and nanotechnology applications, the relationships and behaviour of nanoparticles in the environment and their relationship to plant organisms have been summarized by various authors [33,69,70,71]. Nanoparticles enter the environment naturally or through an engineering approach [72]. An important condition for their entry into food chains is their subsequent solubilization and the formation of an aqueous soluble fraction. Such particles can enter the plant and influence its physiological processes. Recently, a review paper summarizing cytotoxicity of AgNPs has been published [44], and its antibacterial activity against many plant pathogens was ascertained [73]. In the previous study, we studied the effect of green synthesized AgNPs on the morphological parameters of maize plants in a pilot experimental model [74]. It was found that AgNPs prepared by us showed significant antibacterial effects (Sehnal, unpublished results), similar to the study of Doody et al. [75]. In this experimental work, we focused on the detailed study of the effect of AgNPs prepared by green synthesis using extracts from sage (*Salvia officinalis* L.) leaves. 

### 3.1. Preparation of Sage Extract

**Figure 3:**
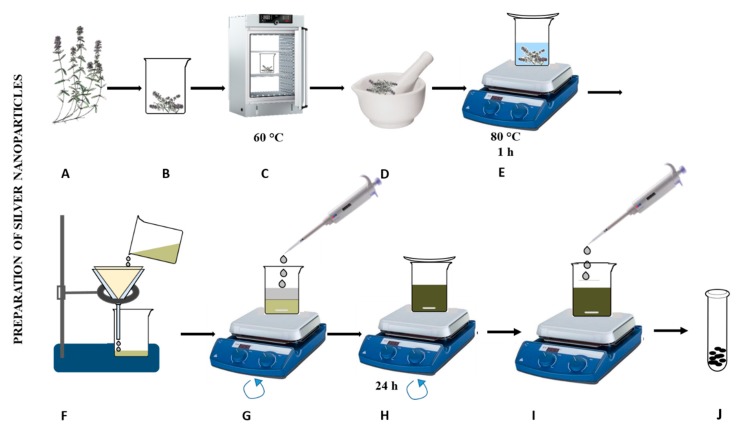
Preparation and characterization of silver nanoparticles (AgNPs)—a simplified scheme for preparing AgNPs. (**A**,**B**) The plant was washed in distilled water, (**C**) dried at 60 °C, (**D**) homogenized to 1-mm particle size, (**E**) and mixed with water and extracted for 1 h at different temperatures (20, 40, 60 and 80 °C). (**F**) The extract was filtered, (**G**) 0.1 M AgNO_3_ (1:1) was added and (**H**) the mixture was stirred for 24 hours. AgNPs have been prepared, (**I**) purified with methanol (**J**) dried and characterized.

Until now, extraction procedures from sage have not been studied in detail to obtain the most suitable properties of AgNPs [60]. So far, we have found that extraction into organic solvents increases the content of active substances, but, deteriorates the properties of the prepared nanoparticles mainly due to the formation of poorly homogenized aggregates (Sehnal, unpublished results). The experimental procedure for the preparation of plant extract and preparation of AgNPs proposed by us is summarized in Figure 3. The plant material was always very gently collected, dried and homogenized to a particle size of 1–2 mm. Subsequently, the plant leachate into the water was prepared. Ultrapure water was chosen as a suitable solvent. The prepared extract was filtered and used immediately to prepare AgNPs. 

Dent el al. [76] found that mass fractions of total and individual polyphenols significantly depend on the type and composition of the extraction solvent and on the extraction temperature. It has further been found that binary solvent systems exhibit much higher efficiency due to their relative polarity compared to mono-solvent systems in the extraction of polyphenolic compounds. For the extraction of polyphenols from dry sage leaves, ethanol or acetone (30%), extraction temperature of 60 °C and extraction time of 30 min were shown to be the most effective [76]. 

In our study, an aqueous extract of *S. officinalis* purified with methanol was used for the preparation of AgNPs (Figure 3). The total protein content of extracts measured using pyrogallol red decreased with increasing temperature applied for extract preparation (from 85 to 55 g/L). Using the biuret test, the highest protein content (93.5 g/L) was found in the extract prepared at 80 °C. The concentration of phenolic compounds in the extracts increased directly in proportion to the preparation temperature of the phytoextract (3–5 mg/L). The content of flavonoids in extracts obtained from *S. officinalis* increased (from 1 up to 5.5 mg/L) with the extraction temperature. Akkol et al. [77] determined the total polyphenolic content in two species of sage (*S. halophila* and *S. virgata*) and the values ranged from 2830 to 21 230 mg per 100 g of extract, depending on the applied extraction solvent. Based on the DPPH method, free radicals (10.5 GAE g/L) were most quenched by sage leaf extracts prepared at 80 °C. This assay is based on the scavenging of DPPH (1,1-diphenyl-2-picrylhydrazyl) by antioxidants, which upon a reduction reaction, decolourize the DPPH methanol solution. The method measures the reducing ability of antioxidants toward the DPPH radical. Using the ABTS method, the ability of extracts to quench free radicals was significantly reduced by increasing the preparation temperature. The colour evaluation of the prepared extracts at different temperatures indicated that the colour intensity decreased with increasing temperature applied for the preparation of the extract. At the highest temperature used, the colour intensity decreased by 15% compared to the lowest preparation temperature.

### 3.2. Synthesis and Characterization of AgNPs

**Figure 4:**
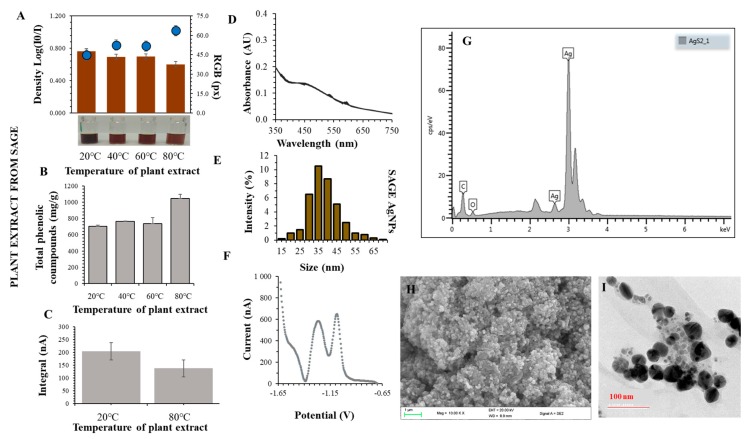
Preparation and characterization of silver nanoparticles (AgNPs)—characteristics of plant extract. (**A**) A typical appearance of AgNPs obtained using a plant extract prepared at 20, 40, 60 and 80 °C. The color intensity was evaluated by ColorTest, (**B**) a typical dependence of total phenolic compounds on the plant extract temperature, (**C**) changes in integrals of thiol compounds of AgNPs prepared at 20 and 80 °C, (**D**) typical Vis spectrum of the prepared AgNPs (scan 0.2 nm), (**E**) hydrodynamic size, (**F**) typical voltammogram of thiol compounds bound to the surface of AgNPs, (**G**) XRD analysis of AgNPs, (**H**) the high-resolution transmission electron microscopic image of AgNPs. (**I**) Polydispersed AgNPs ranged between 2 and 50 nm.

Synthesized AgNPs were prepared by a green synthesis procedure using the above-described aqueous extracts from *S. officinalis*. It is known that the most represented phenolic compounds are rosmarinic acid, caffeic acid, salvianolic acid, sagecoumarin, and sagerinic acid [50,51]. The prepared extract was mixed with 0.1 M AgNO_3_ (1:1, 500 rpm, 25°C). Formation of AgNPs was monitored spectrophotometrically. The signal of around 450 nm in the UV-Vis absorption spectrum confirmed the presence of AgNPs in the mixture [78]. AgNPs were characterized: the hydrodynamic size ranged from 20 to 60 nm, the absorption spectra achieved a maximum peak at 455 nm. Baharara et al. [79] reported a smaller size of AgNPs prepared using *S. officinalis* extract, which was 16 nm. In contrast, Zhang et al. [80] found a much larger size of AgNPs synthesized from the leaf extract of *S. miltiorrhiza*, which was 100 nm. AgNPs formation rate constants were determined by the integration method and were experimentally around 0.3 µM/s/AU. AgNPs were produced most rapidly using an extract prepared at 60 °C. An ideal time was found to produce the largest amount of AgNPs in solution which ranged between 24 and 48 h. The yield of AgNPs produced by green synthesis using sage was 65%. Simple reactions (total phenols, flavones, ability to quench free radicals and total protein content) were used for basic characterization of AgNPs surface properties. Chemical properties: the ABTS method—40–80% of control, the DPPH method—a decrease by 15–55% after 15 min. of radical quenching, total phenols (extract): 700–1200mg GAE/g DW. The TEM analysis showed that the particles were mostly spherical in shape with a size of 50 nm. The SPR method determined the particle size at intervals of 20–60 nm and the zeta potential in the range of −20 to −5 mV. By obtaining the XRD spectrum, we confirmed the presence of silver in the AgNPs and carbon and oxygen on the surface of AgNPs (Figure 4). 

### 3.3. Effect of AgNPs and Ag(I) Ions on Germination

AgNPs can cause various physiological, biochemical, and structural changes in plants. They can cause membrane damage, breakdown of ATP synthesis, and damage to nucleic acids. Nanoparticles cause an increase in oxygen radicals, changes in gene expression, DNA damage or cell death. AgNPs toxicity is observable in germinated plants. There are negative effects on root growth, germination, fresh biomass reduction, and reduction in root elongation and weight. There are also changes in root morphology, stem and leaves. AgNPs modify the expression of several proteins of primary metabolism and cell defence system. An increase in hydrogen peroxide concentration is also important for toxic effects because it affects plant growth and development, and it kills cells. AgNPs have an effect on the mitochondrial membrane. AgNPs toxicity is more noticeable in roots as compared to shoots. The roots are the point of entry of AgNPs into the plant, and the site of defence mechanisms. AgNPs toxicity can be reduced due to aggregation to the plasma membrane and lysozyme-AgNPs interaction [81]. The mechanism of nanoparticle entry into plant tissues and organs is not very clear. A recently published study by Orosa-Puente et. al. suggests potential links to the plant hormone regulatory system [82]. It is known that the soil particles are surrounded by water potential gradients that affect the growth of plant roots and hence their intake of nutrients. The growth of the plant roots is directed towards water due to positive hydrotropism. It was reported that hydropatterning modifies the distribution of root hairs and lateral roots along the root circumference. Recently, the transcription factor ARF7 has been shown to activate the LDB16 gene, inducing the initiation of lateral root formation under water availability conditions. These new findings can help to understand the behaviour of nanoparticles [82,83].

Doody et al. [75] reported the effect of AgNPs on bacterial culture with significant growth inhibition. In addition, they studied the effect of these nanoparticles on maize plants. In this study, the effect of AgNPs on symbiotic *Bacillus subtilis* was further studied. However, the results obtained are still inconsistent and indicate the need for a very intensive study in this area [75]. For food crop mung bean (*Vigna radiata* L.), the effect on growth characteristics on ½ MS agar medium was studied [84]. The shoot, length and weight were reduced (exposure to 50 mg/L). Furthermore, there was an increase in proline, hydrogen peroxide and lipid peroxidation in the roots. In addition, the growth of superoxide in the roots after exposure to AgNPs was demonstrated. The reaction showed changes in the relative mRNA expression of CuZn-SOD, CAT, and APX genes, which indicates antioxidative defence responses of the plants to the AgNP stress [84]. It is also known that metal ions influence the activity of the photosystem and thus the course of photosynthesis [85]. Silver ions released from AgNPs can be expected to subsequently interfere with these key metabolic pathways. In our study, a visual change in the colour intensity of the leaves was noticeable after 24 h in germinated plants of maize, and a significant change in colour was observed after 48 h of exposure. Rooting is a significant problem in horticulture applications; there are still no suitable procedures for a number of woody plants. It is known that silver ions are beneficial in these applications. AgNPs and appropriate plant hormones (IAA, IBA) appear to result in significantly better results. AgNPs probably inhibit soil pathogens and thus roots may form [86]. In wetland plant *Bacopa monnieri*, which is a very fast-growing species in this ecosystem, an increase in protein and sugar levels were observed with lower levels of total phenol content, CAT and POX activities, which is probably related to significant silver ion toxicity [40].

**Figure 5:**
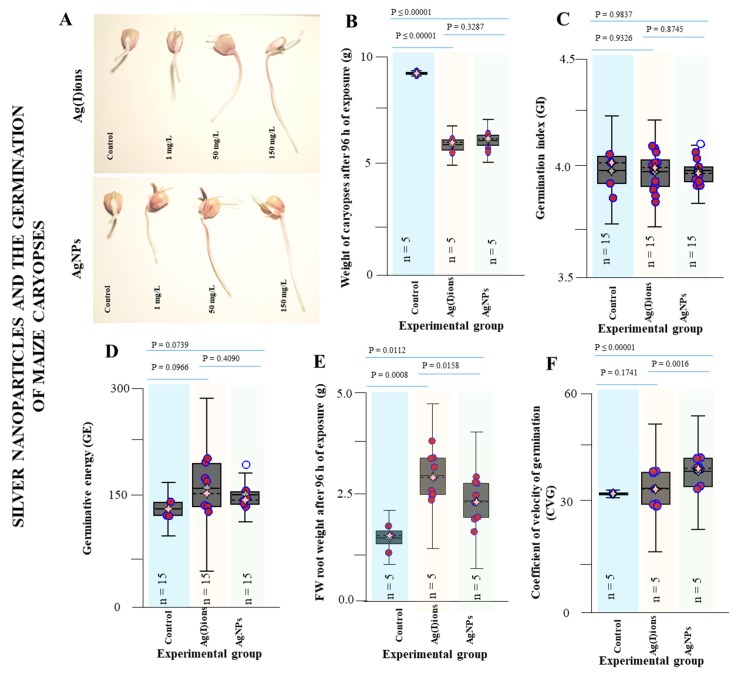
A typical experimental arrangement of Ag(I) ions and AgNPs toxicity tests on germinated plants of maize (10 × 5). (**A**) The germination was evaluated at 96 h. The experiment was run at 25 °C in a cultivation box. The typical appearance of the germinated seeds was evaluated after 96 h. (**B**) Total weight of caryopses after 96 h of exposure. (**C**) Germination index determined after 96 h, (**D**) Germination energy after 96 h, (**E**) Fresh weight (FW) of root after 96 h of exposure, (**F**) Coefficient of velocity of germination. All results presented were determined as the mean of all concentrations (1, 50 and 150 mg/L) tested.

To evaluate the effect of AgNPs and Ag(I) ions on germination, an experiment was prepared as follows: The kernel was placed in a 10 × 5 culture box on a cellulose wadding. Subsequently, the seeds received 100 mL (distilled water-control, AgNPs (1 mg/L), AgNPs (50 mg/L), AgNPs (150 mg/L), Ag(I) ions (1 mg/L), Ag(I) ions (50 mg/L) Ag(I) ions (150 mg/L). The boxes were placed in a cultivation space at 25 °C. After 96 h of exposure, the weights of the individual caryopses exposed to Ag(I) ions and AgNPs were analysed (Figure 5). We found statistically significant differences between the control variant and the application of Ag(I) ions or AgNPs. A statistically significant difference between Ag(I) ions and AgNPs was not detected (mean weight 5.38 vs. 5.61 g). After 96 h of exposure, FWs of primary roots after exposure to Ag(I) ions and AgNPs were analysed. We found statistically significant differences between the control variant (1.68 ± SD 1.36 g) and the application of Ag(I) ions (3.19 ± SD 2.68) and between the control variant and AgNPs (2.58 ± SD 2.12)—(*P =* 0.00112 and *P =* 0.0008, respectively). Also, the difference between Ag(I) ions and AgNPs was statistically significant (*P =* 0.0158). 

The germination characteristics were analysed at 24, 48, (not shown) and 96 h after application of tap water, Ag(I) ions, or AgNP solution. After 96 h of exposure, the experiment was terminated and results from all tested concentrations are shown in Figure 5. Primary roots larger than 3 mm were evaluated. There were no differences between the studied variants in the germination index (Figure 5C). In the germination rate (coefficient of velocity of germination, CVG) analysis, there was a statistically significant difference between the control and the AgNPs variants; on the contrary, there was no difference between the control and Ag(I) variant. The different CVG between the Ag(I) and AgNPs variants was demonstrated by a statistically significant difference (*P =* 0.0016). The germination energy was evaluated from the data obtained. Differences between the Ag(I) and AgNPs variants were not demonstrated (*P =* 0.4090). There was little difference between the control variant and the other two variants—P (Ag(I) ions vs. control) = 0.0966, *P =* 0.0739 (AgNPs vs. control). However, these differences were not statistically significant (Figure 5D). Germination energy increased with increasing the applied concentration of both Ag(I) ions and AgNPs. The primary root length in each individual germinated plant was also analysed. With increasing applied concentration of Ag(I) ions and AgNPs, the average primary root length increased by 19 mm and 12 mm, respectively. Values of the primary root length in individual variants were as follows: the Ag(I) variant—mean length 3.23, median 3.5, minimum 0, maximum 9.0, and SD 1.39 cm, the AgNPs variant—mean length 2.57, median 2.6, minimum 0, maximum 6.2, and SD 0.77 cm, and the control variant—mean length 1.70, median 1.6, minimum 0, maximum 5.3, and SD 0.33 cm. Statistical data analysis revealed a statistically significant difference between control and Ag(I) ions, and between the control and AgNPs. In addition, a statistically significant difference in the primary root length between Ag(I) ions and AgNPs was demonstrated. Significant colour changes on the root surface of germinated plants of maize were observed after 96 h of exposure to both Ag variants tested, especially in the Ag(I) variant, when silver is reduced and directly reacts with proteins. Colour changes in plant tissues were visible in microscopic samples. 

Almutairi et al. [87] reported a positive effect of exposure to AgNPs on maize germination. The germination percentage and germination rate increased with a higher dosage of AgNPs. This could be explained by the fact that nanoparticles are likely to penetrate the seed coat and exert a beneficial effect on the process of seed germination. A possible mechanism is that nanoparticles probably enhance water absorption by seeds [88]. 

**Figure 6:**
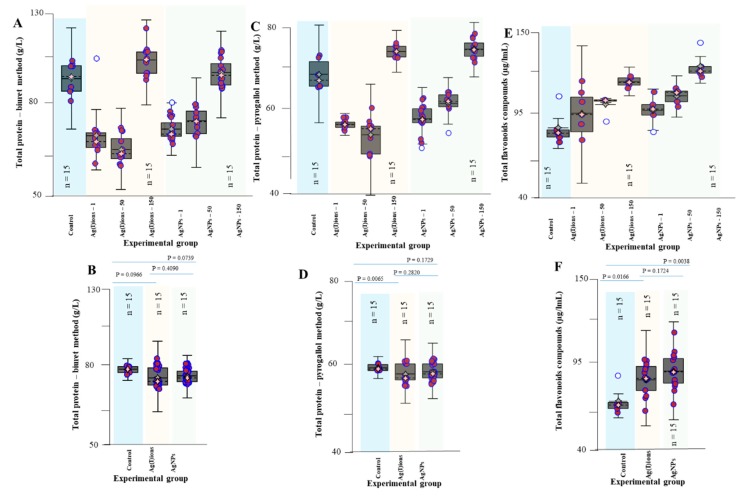
Chemical analysis of the primary roots of germinating maize plants after 96 h exposure to Ag(I) ions and AgNPs. In the study, the following parameters were analysed for all tested concentrations of Ag(I) ions and AgNPs. (**A**) Total protein concentration determined by the biuret method. (**B**) The average total protein ‒ biuret method. (**C**) Total protein concentration determined by the pyrogallol red-molybdate method. (**D**) The average total protein-pyrogallol red-molybdate method. (**E**) Total flavonoid concentration. (**F**) The average value of the total flavonoid concentration.

Total proteins in the roots were analysed using the biuret method after 96 h of exposure to Ag(I) ions and AgNPs (Figure 6). There was no difference between Ag(I) ions and AgNPs. A highly significant difference was found between the control and Ag(I) variant (*P =* 0.0966) and between the control and AgNPs variant (*P =* 0.0739). At applied concentrations of 1, 50 and 150 mg/L, average total protein levels were about 70 g/L and 74 g/L for Ag(I) ions and AgNPs, respectively. However, at an applied concentration of 150 mg/L, the protein level was 88 g/L for Ag(I) ions and 85 g/L for AgNPs. Protein analysis was also performed by the pyrogallol red-molybdate method. The results obtained showed a similar trend in protein content as the biuret method. At applied concentrations of 1 and 50 mg/L of Ag(I) ions or AgNPs, the protein values were about 54 g/L and 56 g/L for AgNO_3_ and AgNPs, respectively. Interestingly, at an applied concentration of 150 mg/L, there was an increase to 64 g/L in both Ag variants tested. A statistically significant difference was found between the control and Ag(I) ions (*P =* 0.0065) but no statistically significant difference was found between the control and AgNPs (*P =* 0.2820). It was found that the average of total flavonoids increased in the Ag(I) ions and AgNP variants with the applied dose and ranged from 100 to 125 µg/mL of the sample, with a slightly higher range of values (from 105 to 135 µg/mL of the sample) in AgNPs. A statistically significant difference was observed between the control variant and Ag(I) ions (*P =* 0.0166) and between the control variant and AgNPs (*P =* 0.0038). No statistically significant difference was observed between the two Ag variants (*P =* 0.1724).

### 3.4. Effect of AgNPs and Ag(I) Ions on Planting Maize Seedlings

**Figure 7:**
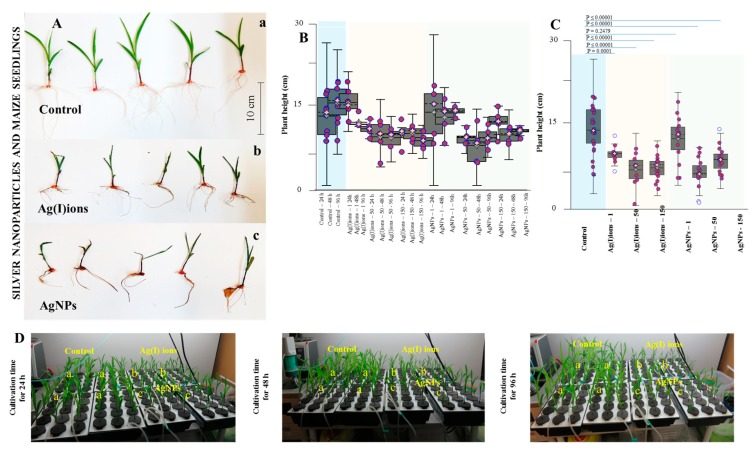
A basic study of the phytotoxic effect of AgNPs on plants of *Zea mays* in hydroponics. (**A**) A typical photo of germinated plants of *Zea mays*—(a) control group, (b) AgNO_3_, (c) AgNPs—after 96 h of exposure to the hydroponic system with tap water. (**B**) A typical course of plant height change at different applied concentrations and exposure time. (**C**) Summarized average plant heights in individual studied variants. (**D**) A typical photo of germinated plants of *Zea mays*—(a) control group, (b) Ag(I) ions, (c) AgNPs—after 96 h of exposure to the hydroponic system with tap water; Ag concentration of 150 mg/L. Plants were collected in triplicate every day and then processed according to the procedure described in the Material and Methods section.

The germinated plants of maize were exposed to Ag(I) ions or AgNPs for 96 h. Growth parameters were evaluated at 24, 48, and 96 h. In the control variant, the average length of the above-ground part of the plants was 15.9 cm. Growth depression after exposure was observed in both Ag tested variants. With a higher concentration of Ag (150 mg/L) compounds and time exposure, growth depression increased. Significant effects were observed in the variant with Ag(I) ions. It was found that at the concentration of 1 mg/L AgNPs, the observed effects on plant growth were statistically insignificant compared to control, and the plants had a similar appearance as the control variant. In the variant with Ag(I) ions, colour changes and a very intense change in leaf turgor were visible. With increasing concentrations of Ag(I) ions and AgNPs, significant growth retardation, discolouration, and leaf tip drying were monitored. The effect of Ag(I) ions or AgNPs on the decrease in plant height was statistically significant when compared with control at all concentration studied with except AgNPs at a concentration 1 mg/L (Figure 7). The total number of leaves on the maize seedlings varied very little for each variant. In the control variant, the average number of leaves was 3.3, in the variant with Ag(I) ions, around 2.7 and in the variant with AgNPs, about 3 leaves per one studied plant (*n* = 150). 

The impact of AgNPs on the growth of different plants can be very different. In contrast to our results, wheat plants (shoots and roots) were relatively unaffected after exposure of their leaves to AgNPs [89]. On the contrary, a study on the effect of biosynthesized AgNPs on *Cucumis sativus* L. seedlings showed that silver significantly reduced the growth which may be due to increased accumulation of silver in plants. The treatment with AgNPs led to a steep reduction in the photo-synthetic performance, total chlorophyll, carotenoids and total protein content and significantly increased oxidative stress parameters [90]. Different plants also respond to different doses of nanoparticles. After applying AgNPs through a foliar spray, the optimum growth promotion and enhanced root nodulation were observed at 50 ppm in *Vigna sinensis*, while improved shoot parameters were recorded at 75 ppm in *Brassica juncea* [89]. 

Hydropatterning changes the distribution of root hairs and lateral roots along the root circumference. It has recently been discovered that the transcription factor ARF7 activates the LDB16 gene, which induces the initiation of lateral root formation when water is available. These recent molecular findings can bring new information on the NPs [82,83]. Application of AgNPs and Ag(I) ions, compared with the control, resulted in highly significant root elongation differences (3.0, 2.6 vs. 1.6 cm, respectively) and a statistically significant increase in root weight (3.18 ± 1.05 g, 2.57 ± 0.65 g vs. 1.68 g, respectively). On the contrary, Yin et al. [91,92] reported that soluble silver in AgNO_3_ exhibited little or no toxic effect on seed germination and plant growth. For both tested variants, the effect on the root system was noticeable. With increasing concentrations of applied amounts of Ag(I) ions and AgNPs, the roots became slightly brown. The overall reduction in plant biomass was due to the plant stress response. The plant probably needed energy and substances to transport and inhibit heavy metals. The plants were exposed to Ag(I) ions and AgNPs (1, 50, and 150 mg/L). Figure 7 shows the dependence of the mean length and weight of plant biomass on the variants tested (control, Ag(I) ions, AgNPs). Plant biomass decreased with increasing concentration and exposure day. Similar phytotoxicity of AgNPs was observed in a study conducted by Yang et al., 2018 [93]. These authors found that wheat grown under different concentrations of AgNPs showed severe phytotoxicity including lower biomass, shorter plant height and lower grain weight. The length of the longest root was measured for each sample. The mean length was 12.7, 8.7, and 9.9 cm in the control sample, in the presence of Ag(I) ions and in the presence of AgNPs, respectively. Another observed parameter was the length of the above-ground portion of the plants. Its mean length was 15.9, 10.8 and 11.6 cm in the control sample, in the presence of Ag(I) ions and in the presence of AgNPs, respectively (growth reduction of about 40%). Subsequently, the average number of roots was counted. There were no differences among group means (control, 7.4; Ag(I) ions group, 7; and AgNP group, 7.5). Also, the fresh root weight was measured. Compared to the control (246.3 mg), there was a decrease of 27.2% in the presence of Ag(I) ions; and in the presence of AgNPs, a decrease by 17.8% was observed. We also monitored the fresh weight of the above-ground portions of plants, which was 377.3 mg in the control; for Ag(I) ions, we recorded a decrease by 47.8%; and for AgNPs, a decrease by 35.6%. Compared to the control, statistically significant differences were found for both tested variants. 

**Figure 8:**
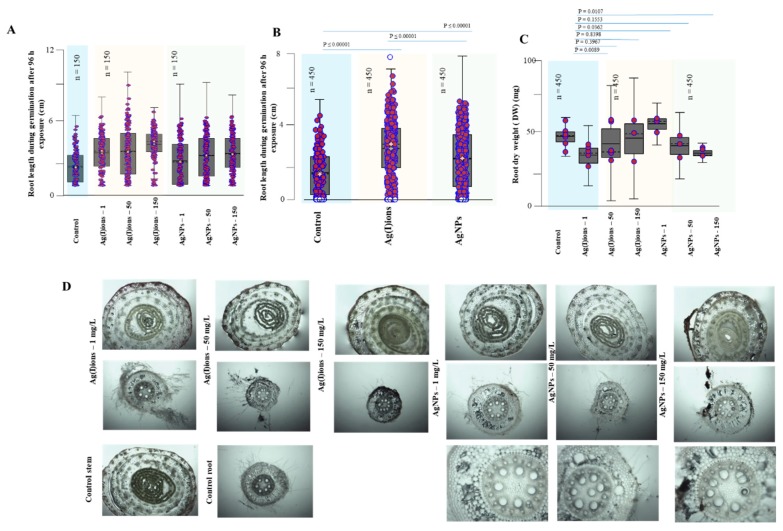
Effect of Ag(I) ions and AgNPs on the root system of germinal plants. (**A**) Root length after 96 h exposure expressed as summary data for each test concentration. (**B**) Summary data for each test group. (**C**) The root dry weight (DW) for each test concentration. (**D**) Changes in the anatomical structure of the stem and root of germinated plants exposed to Ag(I) ions and AgNPs. Plants were collected after 96 h of exposure to hydroponics. Plants were collected in triplicate every day and then processed according to the procedure described in the Material and Methods section.

The total number of roots was determined. In the control variant, the average number of roots was about 7; in the Ag(I) variant, about 6; and in the AgNPs variant, about 7 roots per plant. In the experiment, the DW root (Figure 8C) was evaluated; a more pronounced decrease in weight (40 mg) was observed for the Ag(I) variant (control 53 mg, AgNPs variant 45 mg); however, differences between tested groups were not significant. Changes in the anatomical structure of the stem and root of germinal plants exposed to Ag(I) ions and AgNPs are shown in Figure 8D.

**Figure 9:**
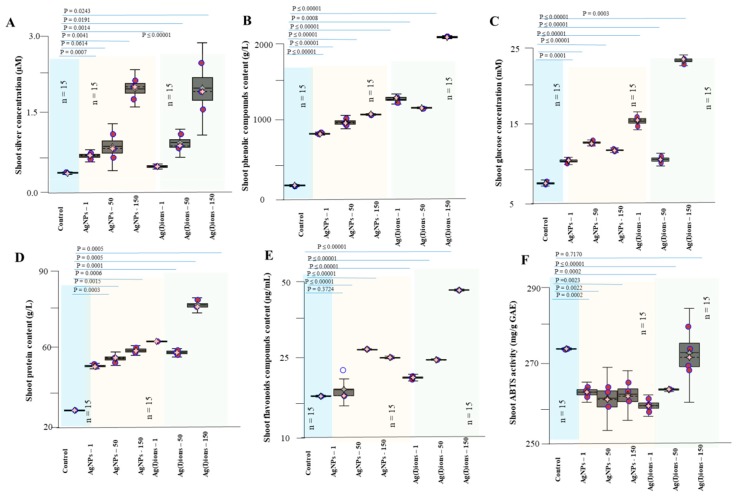
Influence of AgNPs and Ag(I) ions on the above-ground parts of germinated plants. The amount of (**A**) silver ions, (**B**) phenolic compounds, (**C**) glucose, (**D**) total proteins, and (**E**) total flavonoids. (**F**) ABTS activity. Plants were collected after 96 h of exposure to hydroponics. Plants were collected in triplicate every day and then processed according to the procedure described in the Material and Methods section. Data are presented as the average of all experimental data.

**Figure 10:**
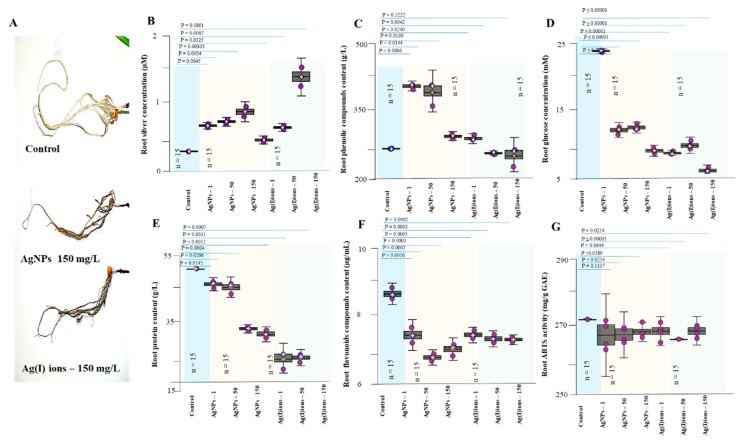
Influence of Ag(I) ions and AgNPs on the root system of seedlings. (**A**) A typical root system appearance after exposure for 96 h. The amount of (**B**) silver ions, (**C**) phenolic compounds, (**D**) glucose, (**E**) total proteins, and (**F**) total flavonoids. (**G**) ABTS activity. Plants were collected after 96 h of exposure to hydroponics. Plants were collected in triplicate every day and then processed according to the procedure described in the Material and Methods section. Data are presented as the average of all experimental data.

The silver concentration was raised in all studied variants (various doses of Ag compounds) and increased in plant tissues with a higher applied concentration (150 mg/L). Noticeable silver levels in plant tissues were found in both the Ag(I) and AgNPs variants. Thus, the ions were transported to the above-ground portion of the plants. On the contrary, it was found that the low concentrations of Ag compounds were probably bound by the plant’s defence mechanisms directly in the roots. High applied concentrations, however, resulted in increased contents in the above-ground portion of the plants (Figure 9). The glucose content in the samples was analyzed by selective reaction with glucose oxidase. There were statistically significant differences between individual studied variants. After treatment of both Ag(I) ions and AgNPs, there was a significant decrease in glucose levels in the roots (8 mM for Ag(I) ions-treated plants, 10 mM for AgNPs-treated plants vs. 23 mM for the controls)—Figure 10D. The results are likely to indicate an effect on plant energy metabolism. The finding is in good agreement with the observed plant appearance after 96 h of exposure. The ability to quench radicals in the extracts obtained was less affected after 96 h of exposure to Ag(I) ions and AgNPs, and a slight decrease in the value was observed (from 290 in the controls to 280 mg/g of GAE in the tested variants)—see Figure 9F. 

**Figure 11:**
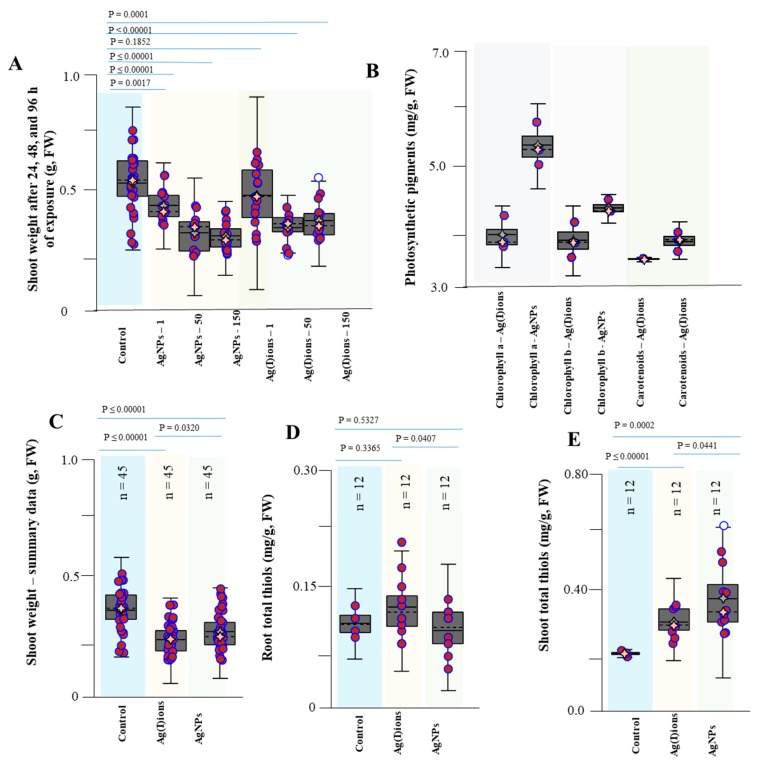
Changes in selected plant parameters exposed to Ag(I) and AgNPs. (**A**) Weight of the above-ground parts of plants as summary data for individual tested variants. (**B**) Changes in photosynthetic pigment contents as average values in individual variants. (**C**) Changes in the total weight of above-ground parts of plants in the Ag(I) ion and AgNPs groups. (**D**) Total thiols in roots. (**E**) Total thiols in the above-ground parts. Plants were collected after 96 h of exposure to hydroponics. Plants were collected in triplicate every day and then processed according to the procedure described in the Material and Methods section. Data are presented as the average of all experimental data.

The weight of the above-ground parts of the plants was affected by the presence of Ag(I) ions and AgNPs. The average weight of control plants was about 0.4 g. Statistically significant growth depression was observed in the Ag(I) variant; the weight decreased from 0.28 g to 0.14 g at its highest applied concentration (150 mg/L). For the AgNPs variant, the weight of the above-ground parts of the plants decreased less vigorously from 0.32 g to 0.20 g (statistically significant) at the highest applied concentration. As is apparent from the experimental data, the weight of the above-ground parts of the plants decreased with the applied dose and the length of exposure. Significant decreases in FW of above-ground parts of plants were detected (control plants 0.38 g, Ag(I)-treated plants 0.19 g, and AgNPs-treated plants 0.24 g; *p* ≤ 0.00001). A significant difference in observed FW weights (*P =* 0.0320) was found between the Ag(I) and AgNPs variants (Figure 11C). Subsequently, the dry matter was obtained from the samples of individual variants. The average dry matter was about 45, 40, and 39 mg in the control, Ag(I), and AgNPs variant, respectively. The observed differences in dry biomass were not as significant as in the case of FW. Thus, the plants significantly changed the content of water present in their tissues.

The toxic effect of AgNPs on primary root length and fresh weight of stem was lower than that of Ag(I) ions. This finding is consistent with the results of the comparative toxicity study of soluble silver (AgNO_3_) and AgNPs on plants performed by Doody et al. [75]. These authors reported that AgNO_3_ toxicity to *Z. mays* was greater than that of AgNPs, as shown by the results of root length and biomass of the seedlings. They also found that *Z. mays* seedlings exhibited significant sublethal effects such as reduced root length and biomass, and hyperaccumulation of Ag in roots. Particularly, the total Ag content in roots increased gradually with elevating exposed concentrations of AgNPs, indicative of enhanced uptake and accumulation of Ag within the plant root [75]. Other authors [90] also found a decrease in the plant growth due to increased accumulation of silver in plants released from AgNPs.

However, AgNPs toxicity depends not only on the concentration but also on the size: smaller particles exhibited a high degree of growth retardation of seeds [91,92]. Plant cell walls function as natural sieves, and particles may have to penetrate them and cell membranes of epidermal layers in roots to enter the xylem in order to be taken up and translocated through stems to leaves [94]. The pore size of plant cell walls is usually only a few nanometers [95]; therefore, smaller AgNPs have relatively stronger effects on plants [96]. Metal ions released from nanoparticles generate oxidative stress within the plant cells which results in free radical-mediated cellular toxicity [97]. A higher amount of metal nanoparticles resulted in a concentration-dependent reduction of gene expression, lower level of photosynthesis and hence retarded the growth of plants [97]. The expected effects of AgNPs phytotoxicity are, in addition to those on the nucleus and mitochondria, also on chloroplasts and thus also on the number of photosynthetic dyes. Furthermore, the amounts of chlorophyll a, chlorophyll b, carotenes, and xanthophylls were calculated by absorbance. We found that the number of photosynthetic dyes increased (control: 675 µg/mL, Ag(I) ions: 827 µg/mL, and AgNPs group: 1261 µg/mL). The increase in the number of photosynthetic dyes of more than 50% in the presence of AgNPs is likely caused by plant defensive reactions due to increased oxidative stress. However, this possible link should be further studied [37]. A statistically significant difference compared with the control was found for both tested variants at a 95% significance level. The amount of thiol compounds in the roots was slightly increased in the Ag(I) variant (0.17 mg/g) and slightly reduced (0.14 mg/g) in the AgNPs variant, compared to the control variant (0.15 mg/g). A significant increase in thiol compounds both in the Ag(I) ions (0.47 mg/g) and AgNPs variants (0.74 mg/g) compared to the control variant (0.11 mg/g) was observed in the above-ground parts of the plants. Subsequently, the weight of the roots obtained was evaluated. There was a statistically significant difference between the control variant (1.68 g) and both silver compounds (AgNPs 3.18 ± 1.05 g, Ag(I) ions 2.57 ± 0.65 g). However, the Ag(I) ion variant was much more similar to the control. Thus, experimental data suggest that AgNPs exhibit different behavior in the germinated plants of maize compared to silver ions. 

Total protein levels were evaluated using the biuret and pyrogallol red-molybdate methods. The amount of total protein in both methods showed a significant decrease after the application of AgNPs and Ag(I) ions (1 and 50 mg/L), indicating an ongoing stress response of a plant likely to bind to proteins, nucleic acids among others. However, at the highest tested concentrations of 150 mg/L, the total protein concentration increased to levels similar to the control group. These changes are likely to be related to the increase in metabolism during the plant’s stress response, overall leading to its total depletion and consequently, death.

## 4. Conclusions

Silver nanoparticles were synthesized using sage leaves by the green synthesis method. AgNPs were biophysically characterized and their phytotoxicity was tested in germinated plants of maize. AgNPs were chemically stable over the course of the experiment and showed an effect on the germinated plants in most of the analysed morphological parameters. These findings should be further investigated.

## Abbreviations

ABTS	2,2′-azino-bis(3-ethylbenzothiazoline-6-sulfonic acid)
AgNP	silver nanoparticles
APX	ascorbate peroxidase
CAT	catalase
DNA	deoxyribonucleic acid
DPPH	2,2-diphenyl-1-picrylhydrazyl
DW	dry weight
FRAP	Ferric Reducing Antioxidant Power
FW	fresh weight
HRP	horseradish peroxidase
IAA	indole-3-acetic acid
IBA	indole-3-butyric acid
LOD	limit of detection
LOQ	limit of quantification
NPs	nanoparticles
P	p-value
POX	plant peroxidase
R	correlation coefficient
RSD	relative standard deviation
SOD	superoxide dismutase
TMB	3,3′,5,5′-tetramethylbenzidine
